# Lettuce‐produced hepatitis C virus E1E2 heterodimer triggers immune responses in mice and antibody production after oral vaccination

**DOI:** 10.1111/pbi.12743

**Published:** 2017-06-09

**Authors:** Jihong Liu Clarke, Lisa Paruch, Mihaela‐Olivia Dobrica, Iuliana Caras, Catalin Tucureanu, Adrian Onu, Sonya Ciulean, Crina Stavaru, Andre Eerde, Yanliang Wang, Hege Steen, Sissel Haugslien, Catalina Petrareanu, Catalin Lazar, Costin‐Ioan Popescu, Ralph Bock, Jean Dubuisson, Norica Branza‐Nichita

**Affiliations:** ^1^ NIBIO‐Norwegian Institute of Bioeconomy Research Ås Norway; ^2^ Institute of Biochemistry of the Romanian Academy Bucharest Romania; ^3^ “Cantacuzino” National Research Institute Bucharest Romania; ^4^ Max Planck Institute of Molecular Plant Physiology Potsdam‐Golm Germany; ^5^ Center for Infection & Immunity of Lille (CIIL) Inserm U1019 CNRS UMR8204 Université de Lille Institut Pasteur de Lille Lille France

**Keywords:** Hepatitis C virus, E1E2 heterodimer, N‐glycosylation, lettuce, edible vaccine, transient expression, lettuce‐produced proteins, molecular farming

## Abstract

The hepatitis C virus (HCV) is a major etiologic agent for severe liver diseases (*e.g*. cirrhosis, fibrosis and hepatocellular carcinoma). Approximately 140 million people have chronic HCV infections and about 500 000 die yearly from HCV‐related liver pathologies. To date, there is no licensed vaccine available to prevent HCV infection and production of a HCV vaccine remains a major challenge. Here, we report the successful production of the HCV E1E2 heterodimer, an important vaccine candidate, in an edible crop (lettuce, *Lactuca* s*ativa*) using *Agrobacterium*‐mediated transient expression technology. The wild‐type dimer (E1E2) and a variant without an N‐glycosylation site in the E2 polypeptide (E1E2∆N6) were expressed, and appropriate N‐glycosylation pattern and functionality of the E1E2 dimers were demonstrated. The humoral immune response induced by the HCV proteins was investigated in mice following oral administration of lettuce antigens with or without previous intramuscular prime with the mammalian HEK293T cell‐expressed HCV dimer. Immunization by oral feeding only resulted in development of weak serum levels of anti‐HCV IgM for both antigens; however, the E1E2∆N6 proteins produced higher amounts of secretory IgA, suggesting improved immunogenic properties of the N‐glycosylation mutant. The mice group receiving the intramuscular injection followed by two oral boosts with the lettuce E1E2 dimer developed a systemic but also a mucosal immune response, as demonstrated by the presence of anti‐HCV secretory IgA in faeces extracts. In summary, our study demonstrates the feasibility of producing complex viral antigens in lettuce, using plant transient expression technology, with great potential for future low‐cost oral vaccine development.

## Introduction

*Hepatitis C virus* (HCV) is an enveloped, single‐stranded, positive‐sensed RNA virus of the *Flaviviridae* family, causing severe liver diseases, which usually progress to fibrosis, cirrhosis and ultimately fatal hepatocellular carcinoma (Luo *et al*., 2014). According to the World Health Organization, approximately 140 million people are HCV‐infected worldwide, with 3–4 million new cases occurring annually (WHO, 2016). The vast majority (80%) of HCV‐infected people live in low‐ and middle‐income countries, the highest prevalence of infection being reported in Africa and the Middle East (Hajarizadeh *et al*., 2013). Innovative anti‐HCV therapies, including the new generation of interferon‐free direct‐acting antivirals (DAAs), have recently become available and offer the possibility to control the epidemic; however, the major drawback of the novel therapeutics is their high cost. In addition, not all HCV‐infected patients can be fully cured and the treatment's side effects are not negligible (Wedemeyer *et al*., 2014). Therefore, development of a prophylactic vaccine against HCV remains a top medical priority, despite serious difficulties, mainly associated with the high genetic diversity of this pathogen (Cashman *et al*., 2014).

The HCV envelope proteins E1 and E2 are type I transmembrane glycoproteins which associate to generate a noncovalent heterodimer, immediately after their processing from the HCV polyprotein by signal peptidase cleavages (Lavie *et al*., 2007, 2015). The E1E2 dimer was shown to induce broad cross‐genotype neutralizing antibodies (nAbs) in chimpanzees and delay the progression to a chronic Hepatitis C infection (Houghton, 2011). Moreover, nAbs were also detected in E1E2‐immunized human volunteers (Stamataki *et al*., 2011), suggesting that the E1E2 dimer represents a promising candidate for future vaccine development. It has reached phase I clinical trials and proved to display good safety and tolerance profiles, inducing strong antibody and lymphoproliferative responses (Frey *et al*., 2010). Interestingly, most of the nAbs are raised against the E2 domain responsible for the interaction with the CD81 viral receptor (Flint *et al*., 1999). In addition, the E1E2 heterodimer exhibits stronger immunogenic properties than either of the two monomers (Dunlop *et al*., 2015). However, the heavy N‐linked glycosylation of the envelope proteins was found to be involved in viral immune evasion, by shielding potentially immunogenic epitopes on the viral surface (Helle *et al*., 2010). Specifically, the E2 N‐glycans were shown to modulate the neutralizing activity of anti‐HCV antibodies (Helle *et al*., 2007). Most of the recombinant E1E2 dimers used in these studies were produced in mammalian cell culture. However, expression in this system is difficult to scale up, requiring for example strict sterile conditions and high costs for GMP, and very costly for commercial use; therefore, cheaper strategies for E1E2 dimer production are urgently needed.

Plant production of biopharmaceuticals, including vaccine antigens and antibodies, is a very attractive alternative production system as plants offer a number of advantages over more traditional systems (Bock, 2014, 2015; Chan and Daniell, 2015; Chan *et al*., 2016; Clarke and Daniell, 2011; Daniell *et al*., 2015). Plant hosts permit the recombinant proteins to be cost‐effectively produced at large scale (due to the very low production costs of plant biomass and the easily scalable production levels, Stoger *et al*., 2014; Ma *et al*., 2015). Also, the risk of contamination from potential human pathogens is very low when using plant‐based production systems (Peyret and Lomonossoff, 2015; Schillberg *et al*., 2013). A recent critical review on the cost‐effectiveness of different vaccine and antibody production systems has shown that plant‐based production systems not only offer the low‐cost advantage, but also several other advantages, such as high safety, production quality and protein folding accuracy when compared with other systems, including mammalian cell cultures, transgenic livestock, yeast and bacterial cultures (Zhang *et al*., 2017). A number of recent advancements and successes in plant molecular farming, such as the ZMapp^™^ antibody against Ebola which saved two lives during the Ebola outbreak, and the cold chain and virus‐free oral booster polio vaccine, have convincingly demonstrated the great potential of plant‐based production systems for biopharmaceuticals (Chan *et al*., 2016; Hiatt *et al*., 2015). Furthermore, the cGMP manufacturing facility available at Fraunhofer USA Center (http://www.fhcmb.org) permits clinical grade plant‐based vaccines and therapeutics to be produced in gram quantities.

Edible plants have additional advantages for the production of therapeutic proteins and vaccines, by facilitating oral delivery, they eliminate the need for expensive fermentation, purification, cold storage, transportation and sterile delivery (Chan and Daniell, 2015; Kwon and Daniell, 2015). Oral delivery of recombinant plant‐produced vaccines is a desirable means of vaccination due to the simplicity and safety of administration. The cost of downstream processing is usually about 80% of the production cost for plant‐made pharmaceuticals and vaccines (Chan and Daniell, 2015; Posgai *et al*., 2017; Shahid and Daniell, 2016). Thus, edible crops offer unique cost advantages. Stability of antigens at room temperature is also a highly desired property of vaccines. Chan *et al*. (2016) have recently reported a cold chain‐independent and virus‐free chloroplast‐made booster vaccine to confer immunity against different poliovirus serotypes, again confirming the great potential of oral vaccines made in edible crops. However, as such examples are still scarce, more research is clearly needed to advance the field of low‐cost oral vaccines produced in edible crops.

In this study, we sought to develop a production system for the HCV E1E2 heterodimer in lettuce (*Lactuca sativa*), an edible crop which has become a promising host for plant‐made oral vaccines and other biopharmaceuticals, using either stable chloroplast transformation or transient expression of genes targeted to the nucleus (Chan and Daniell, 2015). The wild‐type (wt) E1E2 polypeptide and an E2 N‐glycosylation mutant (E1E2∆N6) were successfully expressed in lettuce using *Agrobacterium*‐mediated transient expression. The E1E2 polypeptide was correctly processed in plant cells and the N‐glycosylation pattern was similar to that of the E1E2 dimer produced in mammalian HEK cells (Goffard *et al*., 2005). The antigenic properties of the recombinant HCV proteins were analysed in mice using several immunization strategies, consisting of oral administration of lettuce with or without a previous intramuscular (im) prime using the HEK cells‐expressed HCV dimer. The results demonstrated an early systemic immune response in the mice group receiving the im injection followed by two oral boosts with the E1E2‐expressing lettuce. While immunization performed by oral feeding only resulted in weak serum levels of anti‐HCV IgM for both the E1E2 and the E1E2∆N6 antigens, higher amounts of secretory (s) IgA were detected in the latter case, indicating improved immunogenic properties of the E1E2 heterodimer in the absence of the corresponding N‐linked glycan. In this study, we provide the first evidence that complex viral antigens, requiring host protease processing, post‐translational modifications and an appropriate folding environment to facilitate noncovalent, functional dimerization can be produced in plant cells, while preserving the immunogenic properties of mammalian virus‐derived proteins. The data also highlight the role of particular N‐linked oligosaccharides attached to the E2 glycoprotein in the development of the immune response against HCV. Our study demonstrates the potential of molecular farming and of oral vaccines made in edible crops for future low‐cost vaccine development.

## Results

### HCV E1E2 and E1E2∆N6 expression vectors and transient expression of HCV E1E2 and E1E2∆N6 antigens in *Lactuca sativa*

**Figure 1:**
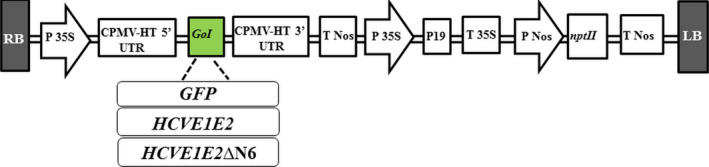
Schematic representation of *HCVE1E2, HCVE1E2ΔN6* and *GFP* gene expression vectors. The E1E2 and E1E2ΔN6 encoding genes were introduced into pEAQ‐*HT*‐DEST1 vector using Gateway cloning technology. RB/LB, right and left borders; 5′UTR and 3′UTR, 5′ and 3′ untranslated regions derived from Cowpea mosaic virus (CPMV); 35S P, 35S promoter; 35S T, 35S terminator; Nos P, nopaline synthase promoter; Nos T nopaline synthase terminator; P19, suppressor of gene silencing; *nptII,* neomycin phosphotransferase II gene conferring kanamycin resistance. GoI, gene of interest.

In order to produce HCV E1E2 and E1E2∆N6 antigens in an edible crop, lettuce, for future oral HCV vaccine development, we designed and constructed two plasmid vectors for expression of the E1E2 and E1E2∆N6 antigens in *L. sativa* cv Veronique (Figure 1). We also constructed a control vector expressing GFP as a reporter gene for protocol optimization and use as a positive control throughout the study (Figure 1). The E1E2 and E1E2∆N6‐encoding transgenes were inserted into the pEAQ versatile transient expression vector developed by Sainsbury *et al*. (2009) using the Gateway^®^ cloning technology (Life Technologies, Carlsbad, USA). All three expression vectors were subsequently transformed into *Agrobacterium tumefaciens* LBA4404 by electroporation as described in the “Materials and Methods” section.

**Figure 2:**
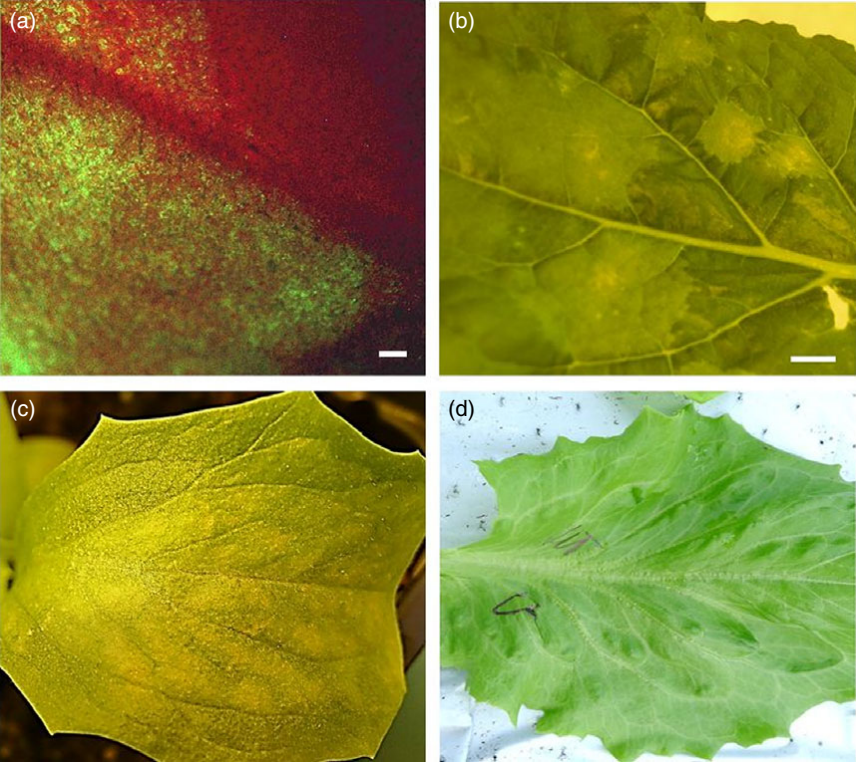
Transient expression of a GFP reporter gene in *N. benthamiana* and of the HCV E1E2 antigens in lettuce. (a) Visualization of GFP expression in *N. benthamiana* 6 days postagroinfiltration (6 dpi) by UV microscopy (bar: 1 mm) and (b) light microscopy (bar 1 cm). (c) Phenotype of HCV E1E2 expressing *Lactuca sativa* at 5 dpi, compared with wild‐type (wt) lettuce as the control (d).

Next, we established a protocol for expression of recombinant proteins in both *Nicotiana benthamiana* and lettuce using the pEAQ‐DEST1/GFP reporter gene‐expressing vector, prior to the production of HCV E1E2 and E1E2∆N6 antigens in lettuce. We optimized the protocol and the technical procedures using the pEAQ‐DEST1/GFP reporter gene expression vector (Figure 2a, b). To produce sufficient amount of E1E2 and E1E2∆N6 antigens required for animal immunogenicity assays, an in‐house assembled vacuum system was applied and HCV E1E2 antigen accumulation 6 days after agroinfiltration (6 dpi) was evident (Figure 2c) when compared with the wild‐type control (Figure 2d). Different operating parameters, such as the power of pressure (bar), and the duration and number of infiltration rounds were investigated to increase the efficiency of the procedure. Various trials have led to the conclusion that vacuum pressure is a crucial parameter for successful infiltration, and this was established at 0.07 bar for lettuce.

### Quantitative analysis of *E1E2* and *E1E2∆N6* mRNA expression in lettuce

The HCV E1E2 and E1E2∆N6 transcript levels were analysed by quantitative real‐time reverse‐transcription PCR (qRT‐PCR) in agroinfiltrated lettuce leaves harvested at different time intervals (dpi). Expression of the reference genes *ACT* and *EIF2A* was used to normalize the values obtained using the ΔΔCq method. The transgene‐derived transcripts were readily detectable in *Agrobacterium*‐infiltrated lettuce at day 6 postinfiltration (dpi) and further increased at 8 dpi, when the highest expression level was reached as shown in Table 1. A considerable amount of RNA transcripts was still detected at 14 dpi (Table 1).

**Table 1:** HCV E1E2ΔN6 gene expression in *Lactuca sativa* cv. Veronique, measured by qRT‐PCR

Sample (lettuce) dpi	Relative normalized expression (fold change)	*P*‐value
0	1.00	
6	38.58	0.000756
8	59.70	0.002639
14	16.95	0.000033

dpi, days postinfiltration.

### Expression of the HCV E1E2 heterodimer in HEK cells and purification

**Figure 3:**
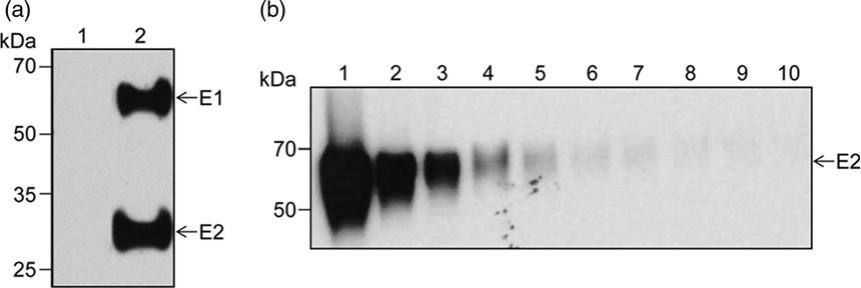
Expression of the HCV E1E2‐encoding gene in mammalian cells. (a) Lysates of control (lane 1) or E1E2‐expressing HEK‐293T cells (lane 2) were subjected to Western blotting followed by sequential detection with anti‐E2 Abs 3/11 and anti‐E1 Abs A4. (b) The GNA‐Sepharose purified E1E2 heterodimer (lane 1) was twofold serially diluted (lanes 2‐10), followed by detection of the E2 polypeptide by Western blotting using the anti‐E2 Abs 3/11.

As a mammalian reference system for the plant‐produced E1E2 heterodimer, the complex was also produced in transiently transfected HEK293T cells (Figure 3a). The purification strategy was based on the property of these N‐glycosylated proteins to bind to lectins with specific affinity for high‐mannose N‐linked glycans, such as the *Galanthus nivalis* (GNA) lectin. Cell lysates were subjected to GNA‐Sepharose affinity chromatography and the high‐mannose proteins were eluted with methyl α‐D‐mannopyranoside. The total protein concentration was determined before Western blot analysis of twofold serial dilutions (Figure 3b). The concentration of the viral antigen was estimated in a semi‐quantitative manner, based on the affinity of the anti‐E2 antibodies (Abs) 3/11 described before (Flint *et al*., 1999) and the sensitivity of the chemiluminescence‐based detection method (pg range). The results indicated a total viral antigen concentration of ~1.6 μg/mL and a purity of ~30% in concentrated samples.

### Expression of the HCV E1E2 heterodimer in lettuce and functional characterization

**Figure 4:**
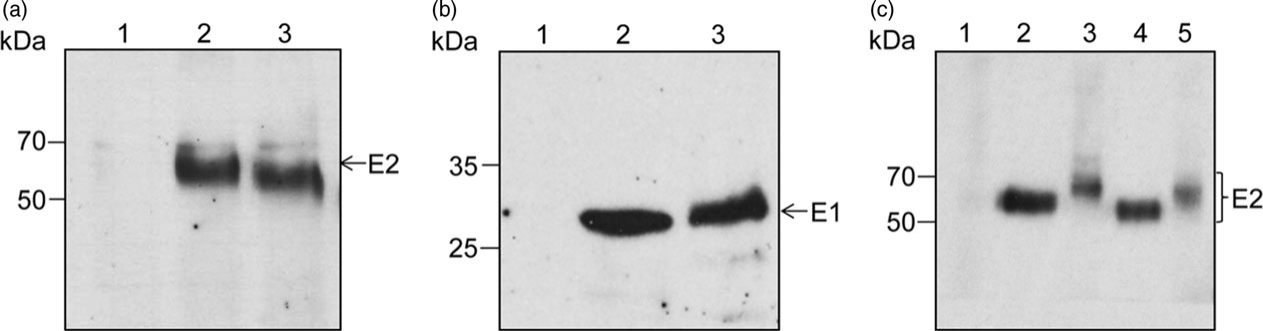
Expression of HCV E1E2 and E1E2∆N6‐encoding transgenes in lettuce. Protein extracts in *Lactuca sativa* control leaves (lane 1), or leaves expressing either the E1E2 (lane 2) or the E1E2∆N6 heterodimer (lane 3) were subjected to immunoblotting and detection with anti‐E2 Abs 3/11 (a) or anti‐E1A4 Abs (b). (c) Analysis as in (a), except that HEK293T cell lysates expressing the E1E2 (lane 2) or the E1E2∆N6 heterodimer (lane 4) were also included, in addition to control lettuce leaf extract (lane 1), and plant extracts containing the E1E2 (lane 3) or the E1E2∆N6 heterodimer (lane 5).

**Figure 5:**
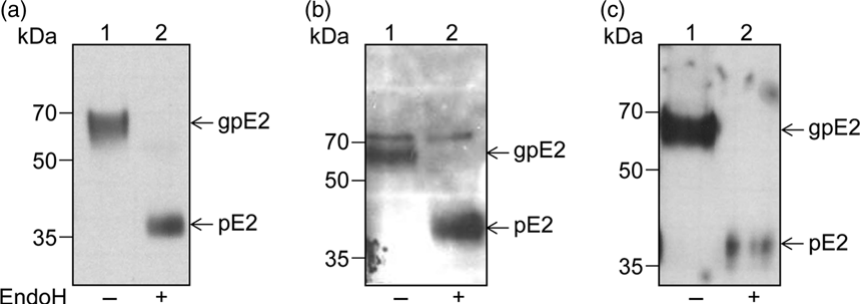
N‐glycosylation of HCV E1E2 and E1E2∆N6 produced in lettuce. Protein extracts from E1E2‐expressing HEK293T cells (a) and *Lactuca sativa* leaves expressing E1E2 (b) or E1E2ΔN6 (c) were treated with EndoH (+) or maintained untreated (−) before Western blot analysis and detection with anti‐E2 Abs 3/11. The glycosylated (gp) and nonglycosylated (p) E2 proteins are shown.

To examine polypeptide processing and determine the level of expression of the E1E2 and E1E2∆N6 viral heterodimers in plant cells, the proteins extracted from lettuce leaves were subjected to Western blot analysis with either E1‐ or E2‐specific Abs. As shown in Figure 4, the wild‐type polypeptide and that of the N‐glycosylation mutant were produced at similar levels in lettuce. The E2 and E1 glycoproteins accumulated at the expected apparent molecular weight of ~70 and ~30 kDa, respectively (Figure 4a, b) suggesting correct processing of the E1E2 viral polypeptide by endogenous processing proteases present in the plant cell, similar to that specific to mammalian cells. The E2∆N6 protein migrated slightly faster than its wild‐type counterpart, consistent with the loss of an N‐linked oligosaccharide (Figure 4a). Interestingly, both lettuce‐expressed E2 proteins displayed a slightly lower electrophoretic mobility when compared to their mammalian cell‐expressed counterparts and appeared as broader bands. This migration behaviour might reflect a more heterogeneous N‐glycosylation or addition of plant‐specific O‐glycans (Figure 4c). To investigate this possibility, the protein extracts were subjected to endoglycosidase H (Endo H) digestion, which cleaves within the chitobiose core of the high‐mannose glycans, but does not cleave complex N‐glycans attached to proteins (Figure 5). As expected, the HEK cell‐produced E2 protein was completely sensitive to EndoH digestion (Figure 5a). The lettuce‐expressed E2 and E2∆N6 polypeptides were also sensitive to EndoH digestion, confirming their glycosylation (Figure 5, panels b and c). However, while EndoH digestion converted the majority of the lettuce‐expressed wild‐type E2 to a protein of similar mobility as the HEK cell‐produced E2, there also appeared to be a minor form that was treatment‐resistant (Figure 5b, upper band). This analysis suggests that some complex sugars may be added during N‐glycosylation processing in plant cells; however, a large envelope protein pool contains high‐mannose N‐linked glycan, as the protein expressed in mammalian cells. Alternatively, this thin upper band could represent a small fraction of unprocessed E1‐E2 polypeptide. The slight reduction in the E2 polypeptide after the EndoH digestion of the E1E2∆N6 lettuce extract is most likely due to precipitation of a fraction of this protein, under the harsh denaturation conditions requested for enzymatic cleavage (Figure 5c).

**Figure 6:**
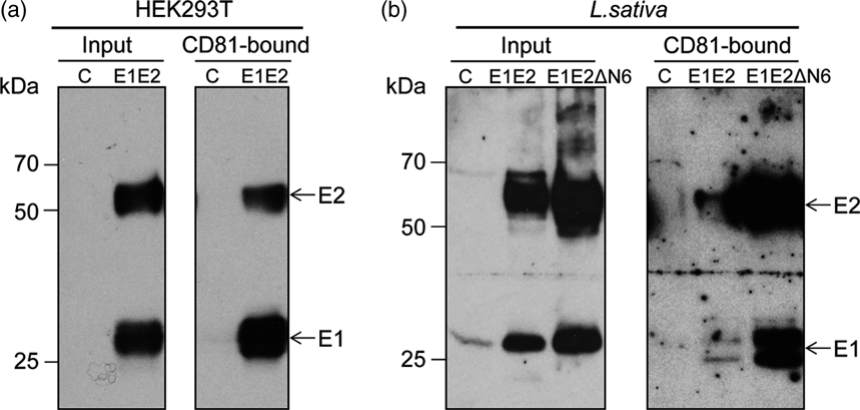
CD81 binding of lettuce‐produced HCV antigens. Protein extracts (input) from HEK293T cells expressing the E1E2 dimer as control (a) and *Lactuca sativa* Veronique leaves expressing the E1E2 or E1E2ΔN6 dimers (b) were reacted with GST‐fused CD81‐LEL adsorbed onto glutathione–Sepharose beads. Lysates and CD81‐bound proteins were analysed by Western blotting and sequentially detected with anti‐E2 3/11 and anti‐E1A4 Abs.

HCV infection critically depends on E1E2 binding to the host cell's CD81 receptor. The highly conserved CD81‐binding regions, localized in the E2 protein, are the major target of a plethora of nAbs. CD81‐E2 interaction indicates exposure of the binding domains within a correctly folded E2 protein. To investigate the formation of the E1E2 heterodimer and the folding state of E2, we performed a pull‐down assay using as bait a recombinant form of the CD81‐LEL (large external loop) which forms the CD81 interaction domain with the HCV E2 protein. Lettuce proteins pulled down by CD81‐LEL were detected by Western blotting. As shown in Figure 6, both E1 and E2 were identified in the pulled‐down samples derived from mammalian (Figure 6a) or plant cells (Figure 6b). This suggests native folding of the CD81‐binding domain of E2, as well as formation of a functional E1E2 heterodimer in plant cells, as E1 does not directly interact with CD81.

### Immunogenicity of lettuce‐derived HCV antigens

To determine the immunogenicity of the HCV antigens expressed in transgenic lettuce, the experimental design included several strategies. In addition to the control group, two groups of mice were fed with either E1E2‐ or E1E2∆N6‐expressing lettuce and two groups of mice were first primed by intramuscular injection with E1E2 purified from HEK293T cells, followed by oral or parenteral boosting with E1E2‐lettuce and HEK293T‐derived E1E2, respectively (Table 2). It is important to note that both oral and parenteral immunizations were performed in the absence of adjuvants, to have a clear evaluation of any antigen‐specific immune response. The kinetics of the anti‐HCV IgM, IgG, IgA Abs accumulation in sera and secretory (s) IgA in faeces was monitored before immunization (day 0) and on days 14, 28 and 42.

**Table 2:** Total antibody positivity rate after different immunization strategies

Group	No. mice	Vaccine and administration		Anti‐HCV Abs	Days post‐prime			
		Prime	2xboost		**0**	**14**	**28**	**42**
				Serum IgM	0/5	0/5	0/5	0/5
0	5			Serum IgG	0/5	0/5	0/5	0/5
				Faeces IgA	0/5	0/5	0/5	0/5
		im	Feeding	Serum IgM	**0/5**	**5/5**	**5/5**	**5/5**
1	5	HEK293T‐E1E2	Lettuce E1E2	Serum IgG	0/5	0/5	0/5	**4/5**
				Faeces IgA	0/5	**4/5**	**4/5**	**4/5**
		Feeding		Serum IgM	0/7	0/7	**1/7**	**2/7**
2	7	Lettuce‐E1E2∆N6		Serum IgG	0/7	0/7	0/7	0/7
				Faeces IgA	0/7	**3/7**	**3/7**	**3/7**
		Feeding		Serum IgM	0/5	0/5	1/5	1/5
3	5	Lettuce‐E1E2		Serum IgG	0/5	0/5	0/5	0/5
				Faeces IgA	0/5	0/5	0/5	0/5
4	5	im	im	Serum IgM	0/5	ND	**5/5**	**5/5**
		HEK293T‐E1E2	HEK293T‐E1E2	Serum IgG	0/5	ND	**5/5**	**5/5**
				Faeces IgA	ND	ND	**5/5**	**5/5**

Im, intramuscular; ND, not determined; bold text, antigen specific reactivity

**Figure 7:**
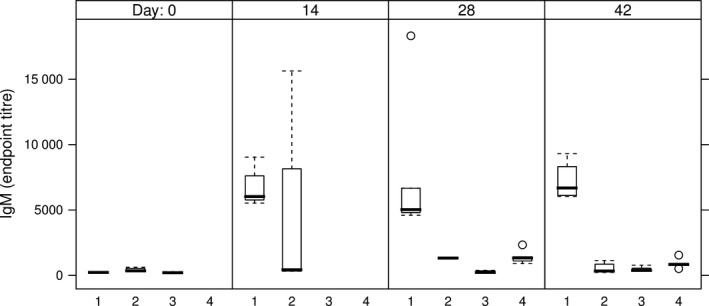
IgM endpoint titres in sera of immunized mice. 1 – intramuscular prime with E1E2 purified from HEK293T cells, followed by two oral boosts with lettuce‐E1E2; 2 – three oral administrations of lettuce‐E1E2∆N6; 3 – three oral administrations of lettuce‐E1E2; 4 – two intramuscular administrations of E1E2 purified from HEK293T cells; endpoint titres were calculated based on a 4‐parameter logistic regression curve fitted to a pool of immune sera, as the reciprocal sample dilution that would result in three times baseline + standard error as derived from the internal standard curve by multiplication.

**Figure 8:**
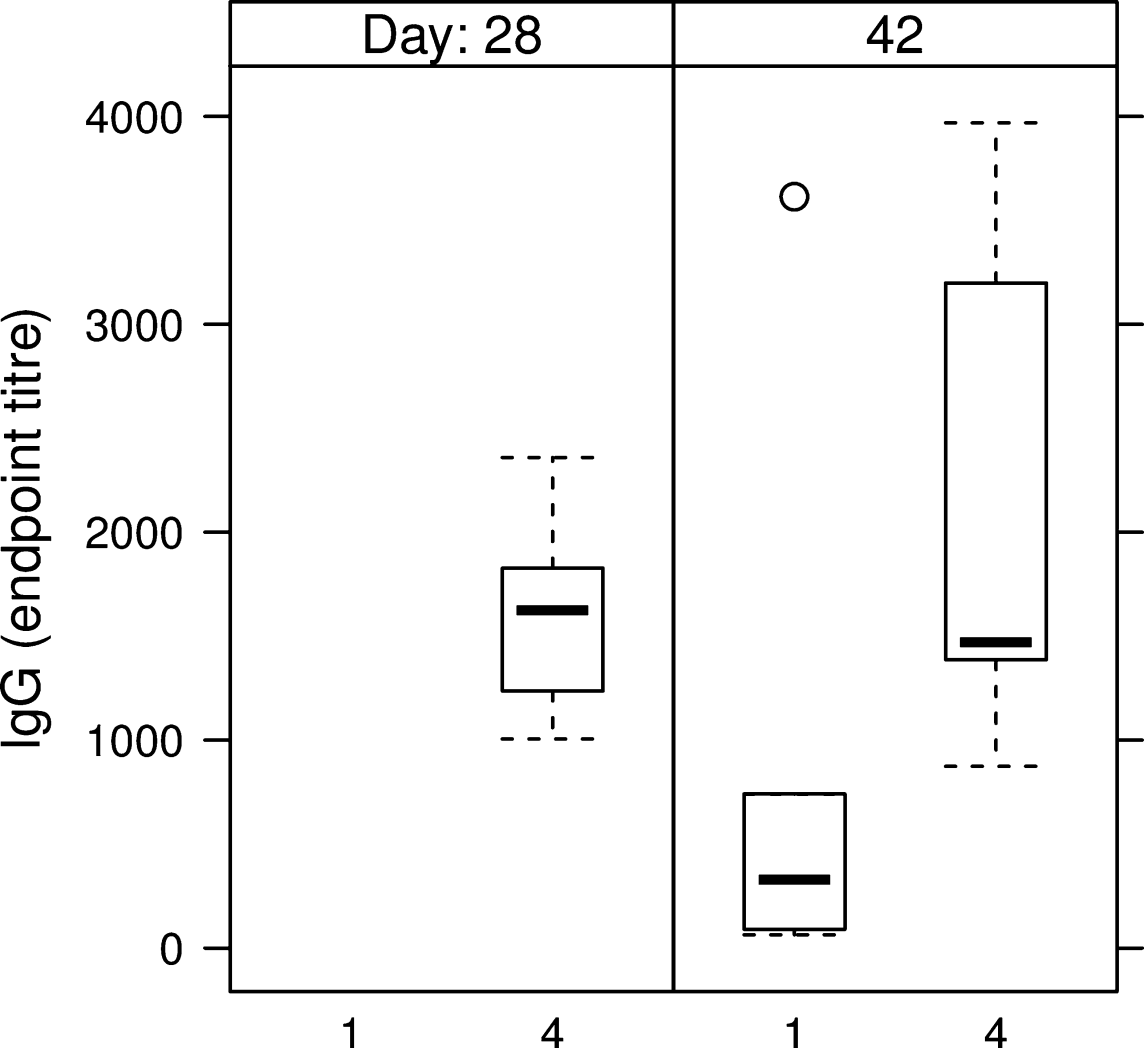
IgG endpoint titres in sera of HEK293T‐derived E1E2 primed mice. 1 – intramuscular prime with E1E2 purified from HEK293T cells, followed by two oral boosts with lettuce‐E1E2; 4‐ prime and boost injection with E1E2 purified from HEK293T cells; endpoint titres were calculated as described in Figure 7.

Anti‐HCV IgM Abs were detected in all mice sera from the groups which received the intramuscular injection (Table 2, groups 1 and 4), indicating the development of a primary immune response (Figure 7). The two groups of mice having received either the E1E2∆N6 or the E1E2 antigen via the oral route only developed HCV‐specific IgM reactivity sporadically and at low level, starting from day 28 (Table 2, groups 2 and 3 and Figure 7). None of these mice developed an anti‐HCV IgG response during the time frame investigated. The development of anti‐HCV IgG Abs was evidenced on day 28 in all mice immunized by injection only (Table 2, group 4) and on day 42 in two of five mice orally boosted with E1E2‐expressing lettuce (Table 2, group 1) (Figure 8).

**Figure 9:**
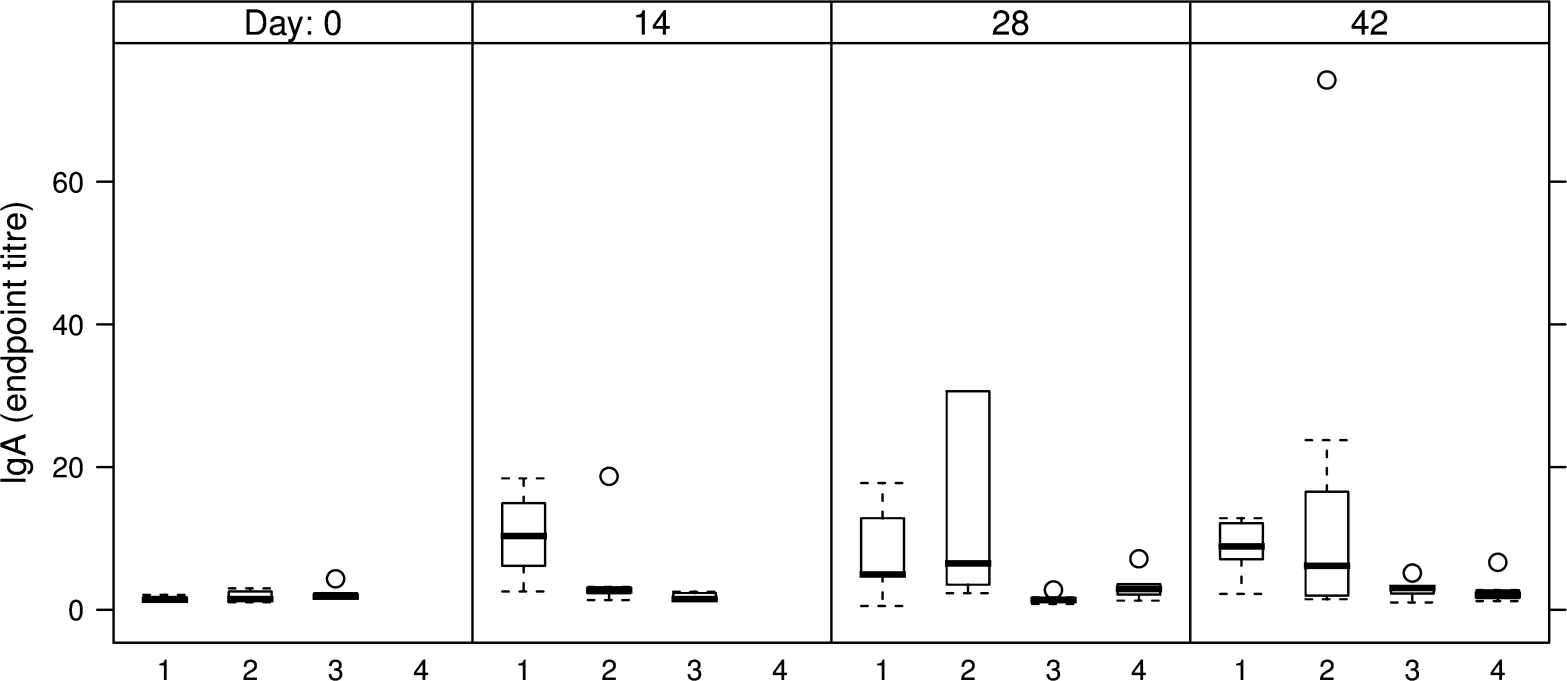
sIgA endpoint titres in faeces of immunized mice. 1 – intramuscular prime with E1E2 purified from HEK293T cells, followed by two oral boosts with lettuce‐E1E2; 2 – three oral administrations of lettuce‐E1E2∆N6; 3 – three oral administrations of lettuce‐E1E2; 4 – two intramuscular administrations of E1E2 purified from HEK293T cells; endpoint titres were calculated as described in Figure 7.

Anti‐HCV IgA Abs were not detectable in sera of either group of mice taken into the study (data not shown). By contrast, HCV‐specific sIgA Abs were detected in faeces extracts of the group of mice orally immunized with E1E2∆N6‐expressing lettuce (Table 2, group 2), but not in the group fed with E1E2 lettuce (Table 2, group 3). Anti‐HCV secretory IgA was also detected in faeces extracts in the mice groups primed by intramuscular injection followed by oral or parenteral boosting (Table 2, groups 1 and 4, respectively). The highest relative concentration of anti‐HCV sIgA was detected in mice orally immunized with E1E2∆N6‐expressing lettuce on day 28, which declined by day 42 (Figure 9, group 2). Interestingly, a comparison between the mice groups primed by intramuscular injection indicated that the relative concentration of anti‐HCV sIgA measured on days 14 and 28 was considerably higher in the group boosted by oral feeding than in the group receiving the intramuscular injection (Figure 9, groups 1 and 4).

## Discussion

HCV vaccine development has turned out to be very challenging because of the high diversity of the HCV genome and the frequent mutations occurring during viral replication. These difficulties are also associated with restricted humoral and cell‐mediated responses against HCV infection, low delivery of potentially protective viral epitopes and poor efficiency of the adjuvants used in different immunization protocols (Garcia *et al*., 2014). A unique feature of HCV infection is the late onset of adaptive immune responses after infection. Production of HCV‐specific T cells appears within 2–5 weeks and seroconversion occurs approximately 6–8 weeks after infection following the initial T‐cell response (Cooper *et al*., 1999; Missale *et al*., 1996). It is well recognized that a strong and broad cytotoxic T lymphocyte (CTL) response is important for HCV clearance (Latimer *et al*., 2014). An effective preventive vaccine should be able to induce strong neutralizing antibodies in addition to a powerful cellular immune response to provide the best long‐term mechanisms for controlling HCV infection (Ippolito *et al*., 2015). Currently, only a few potential HCV vaccines, consisting of recombinant proteins, synthetic peptides, modified vaccinia virus‐based vaccines and DNA‐based vaccines have progressed to clinical trials, with the E1E2 complex being one of the most promising candidate vaccines (Torresi *et al*., 2011; Wong *et al*., 2014).

In the current study, we investigated for the first time the potential of the HCV E1E2 heterodimer as an oral vaccine. Oral vaccines are attractive due to their ability to elicit humoral and cellular immunity via both the mucosal and the systemic immune systems and the potential to eliminate undesired pain and needle‐associated risks from injections which often result in low acceptance (Neutra and Kozlowski, 2006). However, oral delivery is still a challenging immunization route, plagued not only by the possibility of antigen degradation in the gastrointestinal tract (Lugade *et al*., 2010; Silin *et al*., 2007) but also by the lack of efficient oral priming approaches and the risk of inducing tolerance in the absence of host immune system priming (Chan and Daniell, 2015; Kwon *et al*., 2013; Mason *et al*., 2008).

Here, we have demonstrated that the full‐length HCV E1‐E2 polypeptide is efficiently processed by plant‐specific processing proteases resulting in release of mature E1 and E2 proteins. In mammalian cells, these heavily glycosylated proteins undergo a slow and complicated folding process involving acquisition of intramolecular disulphide bridges and noncovalent dimerization. This maturation pathway is strictly dependent on host factors, including molecular chaperones and specific enzymes (Krey *et al*., 2010). The ability of the lettuce‐expressed E1E2 dimer to bind the CD81 receptor and to undergo N‐glycosylation processing similar to that in mammalian cells is strong indication that functional folding of these complex viral proteins occurs in plant cells. Lettuce does not need to be cooked and, therefore, is an ideal candidate edible crop for oral vaccine development. Also, both stable chloroplast transformation and transient expression technologies are well established in lettuce (Ruhlman *et al*., 2007; Lai *et al*.,^2012^; Kwon and Daniell, 2015; Ma *et al*., 2015; Clarke *et al*. unpublished results; Chen *et al*., 2016; current study). Chen and co‐workers have recently demonstrated that a cold chain‐independent plant‐made viral protein 1 (VP1) subunit oral booster vaccine produced in tobacco and lettuce using chloroplast genetic engineering is a promising candidate for future oral booster polio vaccine development (Chen *et al*., 2016). Animal trials have shown that the oral booster VP1 conferred immunity against different poliovirus serotypes. Future clinical trials are hoped to provide further information about the efficacy of this oral booster polio vaccine in humans (Chen *et al*., 2016). However, to date, there are only few reports on oral vaccines produced in lettuce and the functionality of oral antigens in animal trials.

Analysis of the HCV antigen immunogenicity in mice showed that immunization performed by oral feeding only resulted in a relatively weak anti‐HCV IgM response. Interestingly, while anti‐HCV IgG Abs were readily detectable on day 28 in mice primed and boosted by injection, this response was obtained on day 42 when the boost was performed by lettuce feeding. This observation suggests a delayed activation of systemic immunity, possibly as a consequence of oral tolerance acquisition. This hypothesis is also supported by the results showing the absence of anti‐HCV IgG Abs from the sera of mice immunized exclusively by feeding with either E1E2 or E1E2∆N6 dimers.

The ability of plant‐derived vaccines to induce both systemic and mucosal immune response has been previously shown (Kong *et al*., 2001; Walmsley and Arntzen, 2000); however, vaccines delivered by injection, while effective inducers of systemic immunity, are less efficient at inducing mucosal responses (Walmsley and Arntzen, 2000). It is well documented that the gut‐associated lymphoid tissue (GALT) determines adaptive immunity versus tolerance in relation to antigen structure, dosage and duration of exposure (Mowat, 2003; Pamer, 2007). In this respect, a significant difference regarding the faecal levels of sIgA, a hallmark of mucosal immunity activation, was observed between the mice groups fed with lettuce‐derived E1E2 or E1E2∆N6 dimer. Oral immunization with E1E2∆N6‐containing lettuce induced the highest amount of anti‐HCV sIgA Abs, the best response being detected at days 14 and 28, followed by a slow decrease by day 42. This is in agreement with the previous observation that some oligosaccharides attached to the E2 protein may shield potential immunogenic epitopes, and highlights the importance of the Asn532 N‐linked glycan (N6) in this process (Helle *et al*., 2007).

HCV‐specific sIgA Abs were also detected in faeces extracts of mice primed by intramuscular injection. Notably, the Abs titres were significantly higher in the group boosted by oral feeding compared to parenteral inoculation, indicating the importance of the immunization protocol for a successful oral vaccination (Czerkinsky *et al*., 1993; Mestecky *et al*., 2007). As the E1E2 dimer is the current vaccine candidate, both oral and injection administrations of the antigen were performed with this protein. Nevertheless, as the E1E2∆N6 dimer induced higher sIgA titres, when administered exclusively by feeding, it is tempting to speculate that this de‐glycosylated antigen may change the specificity of the antibody response, as compared to the E1E2 protein. Together, these data suggest that association of parenteral with oral immunization may lead to the development of both systemic and mucosal immune responses, which could provide a more effective prophylaxis against pathogens causing chronic diseases such as HCV.

Moreover, its successful transient expression in lettuce demonstrates the potential of plant‐made oral vaccines also in those cases where complex protein maturation pathways are involved. Future studies will aim at determining the optimum parameters for oral vaccination, such as dosage, timing, immunization routes and antigen formulation, and will also include molecular studies on mucosal and systemic immune responses and possible tolerance induction.

## Experimental procedures

### Mammalian cell lines and expression vectors

HEK239T cells (European Collection of Animal Cell Culture, Porton Down, UK) were grown in DMEM (1x) + GlutaMAX™‐I medium (Life Technologies, Carlsbad, USA) containing 10% foetal bovine serum (FBS). Cloning of the full‐length wt E1E2 heterodimer and of the variant containing the mutation Asn532/Gln within the E2 polypeptide (E2∆N6) into the mammalian expression vector phCMVdeltaC was described previously by Helle *et al*. (2007). The resulting expression vectors were further denoted phCMVdeltaC‐E1E2 and phCMVdeltaC‐E1E2∆N6, respectively. The HCV genotype is 1a (isolate H77).

### Expression of the E1E2 heterodimer in HEK cells and purification

HEK293T cells were amplified in T75 tissue culture flasks before being transferred to 1700 cm^2^ ribbed surface roller bottles (Greiner Bio One, Austria) at a density of ~2.5 × 10^7^ cells/bottle, then further grown in a Roll‐In CO_2_ Control incubator (Wheaton). Cells were transfected with phCMVdeltaC‐E1E2 at day 3 postseeding in the presence of 1 μg/mL polyethylenimine (PEI). Cells were harvested at day 3 post‐transfection and lysed in phosphate‐buffered saline (PBS) containing 1% Triton X‐100 and a mixture of protease inhibitors (Santa Cruz Biotechnology, Dallas, USA). Lysates were centrifuged for 10 min at 10 000 ***g*** and the total protein content was determined in clear supernatants using the bicinchoninic acid (BCA) method (Pierce). The supernatants were incubated overnight with *Galanthus nivalis* (GNA)‐conjugated Sepharose (Sigma‐Aldrich, St.Louis, USA) and bound high‐mannose glycoproteins were eluted in the presence of 2 M methyl α‐D‐mannopyranoside (Sigma‐Aldrich, St.Louis, USA). The eluates were extensively dialysed against PBS and concentrated on Amicon50x columns (Merck Millipore, Ireland). Serial dilutions of samples were analysed by sodium dodecyl sulphate‐polyacrylamide gel electrophoresis (SDS‐PAGE) and Western blot with anti‐E2 Abs 3/11 (kindly provided by J.A. McKeating, University of Birmingham, UK).

### Construction of *E1E2* and *E1E2∆N6* plant expression vectors

The E1E2 coding region was excised from the phCMVdeltaC‐E1E2 and phCMVdeltaC‐E1E2∆N6 vectors and inserted into the plant transient expression vector pEAQ‐*HT*‐DEST1 (Sainsbury *et al*., 2009) using a Gateway cloning system provided by Life Technologies (BP&LR Clonase II kit, donor vector pDONR™/Zeo, Invitrogen Carlsbad, USA). The Gateway cloning BP and LR reactions (Karimi *et al*., 2002) were conducted according to the manufacturer's protocol. The GFP reporter gene and the HCV E1E2 and E1E2∆N6 encoding genes flanked by attB1 and attB2 recombination sites generated by PCR reaction (*attB1_HCV*:* GGGGACAAGTTTGTACAAAAAAGCAGGCTATGAATTCCGACCTCATGGG/attB2_HCV*:* GGGGACCACTTTGTACAAGAAAGCTGGGTTTACTCCGCTTGGGATATGA*) were introduced into the donor vector pDONR™/Zeo via BP recombination reaction and thereafter transformed into One Shot OmniMAX™ 2‐T1^R^ Chemically Competent *E. coli*. The generated entry clones from BP reaction were confirmed by sequencing and subsequently introduced into the destination vector pEAQ‐*HT*‐DEST1 (kindly provided by Prof. Lomonossoff's laboratory and described by Sainsbury *et al*., 2009) by LR reaction generating pEAQ‐*HT*‐DEST1/GFP, pEAQ‐*HT*‐DEST1/E1E2 and pEAQ‐*HT*‐DEST1/E1E2∆N6 transient expression vectors.

### *Agrobacterium*‐mediated transient expression in lettuce

The plant expression vectors pEAQ‐*HT*‐DEST1/GFP, pEAQ‐*HT*‐DEST1/E1E2 and pEAQ‐*HT*‐DEST1/E1E2∆N6 were introduced into the ElectroMAX™ *Agrobacterium tumefaciens* LBA4404 cells (Invitrogen Carlsbad, USA) by electroporation using BTX ECM 630 electroporator (BTX‐Division of Harvard Apparatus, USA, 2.0 kV, 200 Ω and 25 μF as described by Clarke *et al*., 2008). The *Agrobacterium* LBA4404 harbouring the expression vectors were cultured on LB medium supplemented with 50 mg/L kanamycin and incubated at 28 °C for 72 h. The transformed clones were verified and confirmed by colony PCR using the gene‐specific‐attB1/attB2 primer pair shown above (attB1_HCV/attB2_HCV).

For agroinfiltration, the *Agrobacterium* harbouring pEAQ‐*HT*‐DEST1/GFP, pEAQ‐*HT*‐DEST1/HCVE1E2 and pEAQ‐*HT*‐DEST1/HCVE1E2∆N6 plasmids were cultured and prepared for the agorinfiltration basically as described by Sainsbury *et al*. (2009) and Saxena *et al*. (2016) with minor modifications including the establishment and optimization of our in‐house vacuum‐based agroinfiltration protocol. Four‐six‐week old lettuce plants grown in the growth chamber under a photoperiod of 16‐h light and 8‐h dark with a temperature of 22 °C were subjected to agroinfiltration, using a 2‐mL syringe without needle on the underside of the leaf, basically as described by Sainsbury *et al*. (2009). After the agroinfiltration, the lettuce plants were further grown in the confined S2 safety level growth chamber with the same light and temperature conditions.

Agroinfiltration at a large scale (>30 plants) was carried out by an in‐house assembled vacuum system. Due to the different plant characteristics, the different vacuum conditions were developed and optimized for *Nicotiana benthamiana* and lettuce, respectively, with pressure ranged from 0.01 to 0.1 bar; for 1 and 2 min; repeating one or two times.

### Quantitative analysis of *E1E2* and *E1E2∆N6* expressions in lettuce by qRT‐PCR

Leaf samples harvested at different time intervals (dpi) were submerged immediately in liquid nitrogen and total RNA was isolated using Spectrum™ Plant Total RNA kit (Sigma‐Aldrich, St.Louis, USA). An additional clean‐up to remove any trace DNA left in the samples was performed using Ambion Turbo RNA Purify kit (Ambion Austin, USA). The quality and concentration of the final yielded RNA was evaluated by Bioanalyzer (Agilent Technologies Santa Clara, USA). The cDNA was synthesized from 1.0 μg RNA using a cDNA synthesis kit (Bio‐Rad, Hercules, USA). The qRT‐PCR reactions were carried out on CFX96 real‐time system (Bio‐Rad, Hercules, USA) using SsoAdvanced SYBR Green SuperMix (Bio‐Rad, Hercules, USA). A 10‐ng cDNA was applied as template into 20 μL qPCR in addition to 0.8 μm primers (HCV/N6_F: TTCAGGCTGTCCTGAGAGGT and HCV/N6_R: CCACAAGGTCTTGGAGGGTA). The endogenous reference genes actin and elongation factor were used (ACT_F: AGGGCAGTGTTTCCTAGTATTGTTG and ACT_R: CTCTTTTGGATTGTGCCTCATCT) and *EIF2* (EIF2A_F: TAGGCGAGTGGAGAAGCATT and EIF2A_R: GTAGAAACAGCAACAGGCAAA). The qPCR thermal cycle programme was with initial denaturing at 95 °C for 6 min followed by 40 cycles at 95 °C for 15 s, 61 °C for 15 s and 72 °C for 45 s. Gene expression analysis was performed using the built‐in CFX Manager (Bio‐Rad, Hercules, USA). Normalized expression ΔΔCq was used to estimate the transcript level using the differences of Ct (threshold cycle) value between endogenous reference genes and target gene.

### Antigen extraction from pEAQ‐*HT*‐DEST1/E1E2 and pEAQ‐*HT*‐DEST1/E1E2∆N6 expressing lettuce plants and N‐glycosylation analysis

Lettuce leaves were ground in liquid nitrogen. The ground material was then homogenized in 5 volumes of extraction buffer containing 20 mm Na2HPO4, 0.15 m NaCl, 20 mm Na ascorbate, 0.5% Triton X‐100 and a protease inhibitor cocktail (Santa Cruz Biotechnology, Dallas, USA), pH 7, for 1 h, on ice. The homogenate was centrifuged at 1000 ***g***, for 5 min, at 4 °C. The supernatant was collected, and the total protein content was determined using the BCA method. To determine the N‐glycosylation status of the viral antigens, samples were further treated with EndoH (New England Biolab, UK) following the protocol supplied, then subjected to SDS‐PAGE and Western blotting with either anti‐E1 Abs A4 (kind gift from H.B. Greenberg, VA Medical Center, Palo Alto, California) or anti‐E2 Abs 3/11.

### CD‐81 pull‐down assay

A recombinant large extracellular loop of human CD81 (CD81‐LEL) fused to glutathione S‐transferase (GST) was adsorbed onto glutathione–Sepharose beads (GE Healthcare), as previously described (Helle *et al*., 2007). Nontransformed lettuce lysates (negative control) or extracts containing the E1E2 and E1E2∆N6 proteins were added to 50 μL of beads and incubated overnight at 4 °C. The beads were washed two times with PBS containing 1% Triton X‐100 then resuspended in Laemmli buffer containing 200 mm Tris‐HCl (pH 6.7), 0.5% SDS, 10% glycerol and 100 mm β‐mercaptoethanol. Samples were boiled and separated by SDS‐PAGE followed by Western blotting and detection with either anti‐E1 or anti‐E2 Abs. Lysates of E1E2‐expressing HEK cells were used in the experiment as positive control.

### SDS‐PAGE and Western blotting

Protein samples were heat‐denatured in Laemmli buffer and run on SDS‐PAGE (10% polyacrylamide gels). Separated proteins were further blotted onto nitrocellulose membranes using a semi‐dry blotter (Bio‐Rad, Hercules, USA). The membranes were blocked with 5% nonfat milk in PBS and incubated with the rat anti‐E2 3/11 Abs or the anti‐E1 A4 monoclonal Abs (both diluted at 1/2000 in 5% nonfat milk in PBS with 0.1% Tween). Goat anti‐rat‐HRP and rabbit anti‐mouse HRP‐conjugated antibody (Santa Cruz Biotechnology, Dallas, USA dilution 1/10 000) were used as secondary Abs. The protein bands were visualized using an ECL substrate (Thermo Scientific Waltham, USA). The amount of HCV antigens was determined based on serial dilutions of known concentrations of HEK293T‐produced E1E2 dimer. The level of expression of each of the HCV antigens was estimated at 90 ng/g leaf material.

### Animals and immunization

BALB/c female mice, 6–8 weeks of age, were purchased from Charles River Laboratories (INNOVO Ltd., Hungary) and housed with free access to standard chow and tap water. All animal care was in accordance with standards set forth in the Council Directive 86/609/EEC, and the mice experimentation was approved by the Internal Ethics Committee of the “Cantacuzino” Institute. The mice were randomly assigned in five groups: four groups of five animals including nonimmunized mice and one group of seven animals (Table 2).

Three of the groups received the antigens three times at 1‐week intervals, either by the oral route only or by parenteral priming followed by oral antigen boosting. Oral immunization consisted in individual feeding of mice, overnight, with a pellet obtained by mixing 10 g lettuce powder (containing ~900 ng of either E1E2 or E1E2∆N6 antigens) obtained by liquid nitrogen pulverization, with ~2 g standard chow powder. Priming was done with 50 μL PBS suspension containing 5 μg of HEK293T cells‐produced E1E2 antigen, given by intramuscular injection. One group received two injections with 2.5 μg of HEK293T cells‐produced E1E2 antigen at a 14 day interval, at the same volume per dose (Table 2).

Blood samples were collected by retro‐orbital bleeding prior to priming, at day 14 before the third oral antigen boost and at days 28 and 42 postimmunization. Following clotting, samples were centrifuged at 14 000 ***g*** for 10 min at room temperature and the resulting sera were stored at −80 °C until further analysis. Faecal pellets were also collected from each mouse, at the same intervals, and diluted in PBS containing 0.02% sodium azide (Sigma‐Aldrich, St.Louis, USA), to a final concentration of 10 mg dry matter/mL of PBS. Diluted faecal pellets were homogenized and centrifuged at 14 000 ***g*** for 10 min. Clear supernatants were collected and stored at −80 °C until assayed.

### Enzyme‐linked immunosorbent assay (ELISA)

Flat‐bottom 384‐well MediSorp plates (Sigma‐Aldrich, St.Louis, USA) were coated with 0.24 μg/mL of HEK293T cell‐produced E1E2 antigen, in PBS, overnight at 4 °C. The plates were washed five times with PBS containing 0.05% Tween 20 (Sigma‐Aldrich, St.Louis, USA), blocked with PBS containing 5% nonfat dry milk (Bio‐Rad, Hercules, USA), for 1 h at room temperature and further washed 5 times with PBS containing 0.05% Tween 20. Serum samples were twofold serially diluted (1:200–1:6400) in PBS containing 5% nonfat dry milk then added to the antigen coated plates (40 μL/well) and incubated for 2 h at room temperature. Faecal suspensions (40 μL/well) were twofold serially diluted (1 : 2–1 : 8) as above. The plates were washed five times with PBS containing 0.05% Tween 20, before incubation with HRP‐conjugated goat anti‐mouse IgM/IgG/IgA adsorbed against human immunoglobulins (Southern Biotech), diluted 6000‐fold in PBS containing 5% nonfat dry milk, for 2 h at room temperature. The faeces samples were incubated with HRP‐conjugated goat anti‐mouse IgA only. The plates were washed five times before addition of the TMB substrate (R&D Systems) and further incubated for 20 minutes at room temperature. Reactions were stopped with 20 μL/well of 1M H_2_SO_4_ and absorbance was measured at 450 nm using a Tecan Infinite M1000 microplate reader.

### Data normalization and processing

To allow normalization across ELISA plates, an internal standard was produced by pooling all the immune sera from the same experiment in one control sample. Three internal standards were also produced from immune faeces suspension collected at day 14 from the E1E2/E1E2∆N6 antigens‐immunized groups and the control group, respectively. Twelve serum and faeces serial dilutions starting from 1/100 and 3/4, respectively, were used as internal standard on each plate and a 4‐parameter logistic regression model was fitted to the measured values. Sample dilutions falling within the standard range, with an estimated error <30%, were averaged by median. Endpoint titres were calculated as the reciprocal sample dilution that would result in three times baseline + standard error as derived from the internal standard curve by multiplication. All calculations were performed in R version 3.1.1 (Team, 2014) using the calibFit 2.1.0 package (Haaland *et al*., 2011).
